# Best Location and Reader Role in Usage of Emergency Manuals During Critical Events: Experienced Emergency Manual Users’ Opinion

**DOI:** 10.7759/cureus.4505

**Published:** 2019-04-20

**Authors:** Jeffrey Huang, Kyle Sanchez, Jiayan Wu, Andrey Suprun

**Affiliations:** 1 Anesthesiology, University of Central Florida College of Medicine, Orlando, USA; 2 Medicine, University of Central Florida College of Medicine, Orlando, USA; 3 Anesthesiology, Zhongshan Traditional Chinese Medicine Hospital, Zhongshan, CHN

**Keywords:** emergency manuals, location, reader, survey

## Abstract

Introduction

Emergency manuals (EM) are an essential component of the response to critical events by healthcare providers, and there is strong evidence to support the benefits of utilizing EMs in crisis management. Despite the widespread utilization of EMs by providers, there is currently no national standardized protocol for EM usage, accompanied by a deficit in research on EM usage. To develop a protocol for EM utilization, factors such as the best location for EMs to be placed and the EM reader role must be determined.

Methods

Anesthesiologists with experience in EM use from seven hospitals participated in a survey questionnaire that was distributed to all participants through WeChat using the WenJianxen software. Survey response numbers were converted into percentages and were used to summarize the categorical variables.

Results

Results showed the best location of EMs used during critical events was in the anesthesia station of the operating room and that the preferred reader of EMs during critical events is the senior physician.

Conclusion

Our study suggests that placing EMs within close reach of the health care providers may be most efficient and that the reader of EMs should be the leader of the clinical team. These results may be applicable to the development and implementation of a national protocol for EM usage.

## Introduction

Emergency manuals (EMs) have been implemented with broad generalizability in many industries that have the risk of high hazards and critical events, including nuclear power, military, and aviation, where they primarily serve to guide the cognition of workers during a variety of emergency settings. Over time, the concept of EMs and guided cognition has been translated to medicine, where it is especially beneficial to fields like emergency medicine, anesthesiology, surgery, and critical care. 

As demonstrated in a study, operating room (OR) EMs allowed healthcare providers to respond to and resolve crises more efficiently [1]. These EMs are books that consist of a sequence of current, medically established guidelines utilized in response to specific critical events [2]. It must be emphasized that although many of these clinical emergencies are uncommon, the prompt recognition of such crises followed by a precise, guideline-based response may often tip the balance between life or death toward survival when these events do occur [2].

The clinical advantage of EMs and other cognitive aids has been demonstrated by several publications. These cognitive aids have been shown to benefit the outcome of many types of critical events, and most providers stating that EMs helped their team deliver better care [3]. With the aid of an EM, a four-month-old was saved from malignant hyperthermia [4]. Similarly, a 44-year-old male who developed bronchospasm during a left hepatic lobectomy and cholecystectomy was rapidly and effectively managed via the utilization of an EM [5]. 

The advantage of EMs extends far beyond the U.S. healthcare system. To facilitate the implementation of anesthesia EMs in China, Stanford Operating Room Emergency Manuals, Harvard Ariadne Lab Operating Room Crisis Checklists, and Society of Pediatric and Anesthesia Pedicrisis Critical Events Cards were translated into Chinese [6]. Approximately one year following publication, surveys were distributed to Chinese anesthesia providers from multiple institutions. The survey results revealed a strong association between higher levels of EM use and anesthesiologist participation in simulation training, EM group studies and self-review [6]. Thus, Simulation Wars was created in China to encourage the providers to participate in simulation training [7]. For this competition, the participating hospitals created a self-written and a self-directed video that illustrated the application of the Stanford Operating Room Emergency Manuals to an anesthesia-related critical event. During the final round, they performed in-person crisis management skills utilizing EMs [7]. Approximately one year following the competition, this study found increased use of EMs in real-life crises among providers who participated in a simulation competition [8].

The EM “reader” plays a significant role in the crisis management process. It has been demonstrated that a dedicated EM reader significantly increases the rate of completion of vital actions during simulated crises. While the reader role in clinical care is valuable when successful, it is often reliant on other outside factors, including prior experience with the role and the degree of team member contribution. During clinical critical events, a reader role must be triggered by either a leader that delegates or another clinician that volunteers to be a reader [2]. To our knowledge, it remains unclear who should assume the reader role during the crisis management. 

Today, EMs are established as an important part of OR culture, which help in optimizing response to acute patient situations perioperatively. Despite the growing acceptance of EM incorporation into OR culture, there has been little work investigating the best methods to incorporate crisis checklists and emergency manuals into everyday practice. Many questions remain unanswered: (1) Where should they be located? (2) Who should be the reader during an emergency? The answers to these questions are critical for standardizing EM usage and maximizing its efficiency in multiple hospitals and clinical settings. The purpose of this survey study was to identify the best location for EMs within the OR and decide who should be the reader of the EM in acute perioperative situations, as determined by the opinion of anesthesiologists.

## Materials and methods

This study was approved by a local hospital authority (Zhongshan Traditional Chinese Medicine Hospital). Seven hospitals participated in the Simulation Wars competition 2017. The participating anesthesiologists have EM in their operating rooms, self-review EMs, have participated in the simulation training, and have experience using EM during critical events. These participants, thus, were classified as experienced EM users. We invited them to participate in the survey study.

For this study, ‘self-review’ was defined as studying EM by oneself. Information on the total number of anesthesiologists in each participating department was collected. To protect the confidentiality of physicians, we did not collect identifying information from respondents. No patient-identifiers were collected. The survey questionnaire was adapted from the survey used in the Stanford study and modified by the author. It was then distributed to all respondents through WeChat using the WenJianxen software.

To maximize total responses, the survey did not contain any open-ended questions. Survey questions included the five-point Likert scale (strongly disagree to strongly agree), yes or no boxes, and multiple-choice answers. No monetary compensation was provided for any kind of participation in this survey. Emergency manual use reports for clinical critical events, by event type, were listed as “check all that apply.”

Survey response numbers were converted into percentages and were used to summarize categorical variables. 

## Results

The data from the seven hospitals that participated in this survey were combined. The survey response rate is 114/133 (85.7%). Overall, there was spread among the participants in their positions and the amount of working experience. A total of 114 participants were recruited to complete our survey, which included 49 residents, 35 attendings, and 30 chief physicians. Twenty-eight percent of participants had less than five years of working experience, 28.95% had five to 10 years of working experience, 28.07% had 10 to 20 years of working experience, and 14.91% had over 20 years of working experience.

The access and frequency of EM usage are shown in Table 1. While 68.42% of respondents stated they have a digital version of EM in their smart phone, only 47.71% stated they reviewed the EM at least once last year. However, 90.35% of the respondents stated they used EM during a critical event last year with the majority having used EM one time.

**Table 1:** Access and frequency of emergency manual usage in participants EM, emergency manual

Parameter	Percentage of participants which fulfill parameter
Digital EM on smartphone	68.42%
Reviewed EM last year	47.71%
Used EM during a critical event last year	90.35%

**Figure 1:**
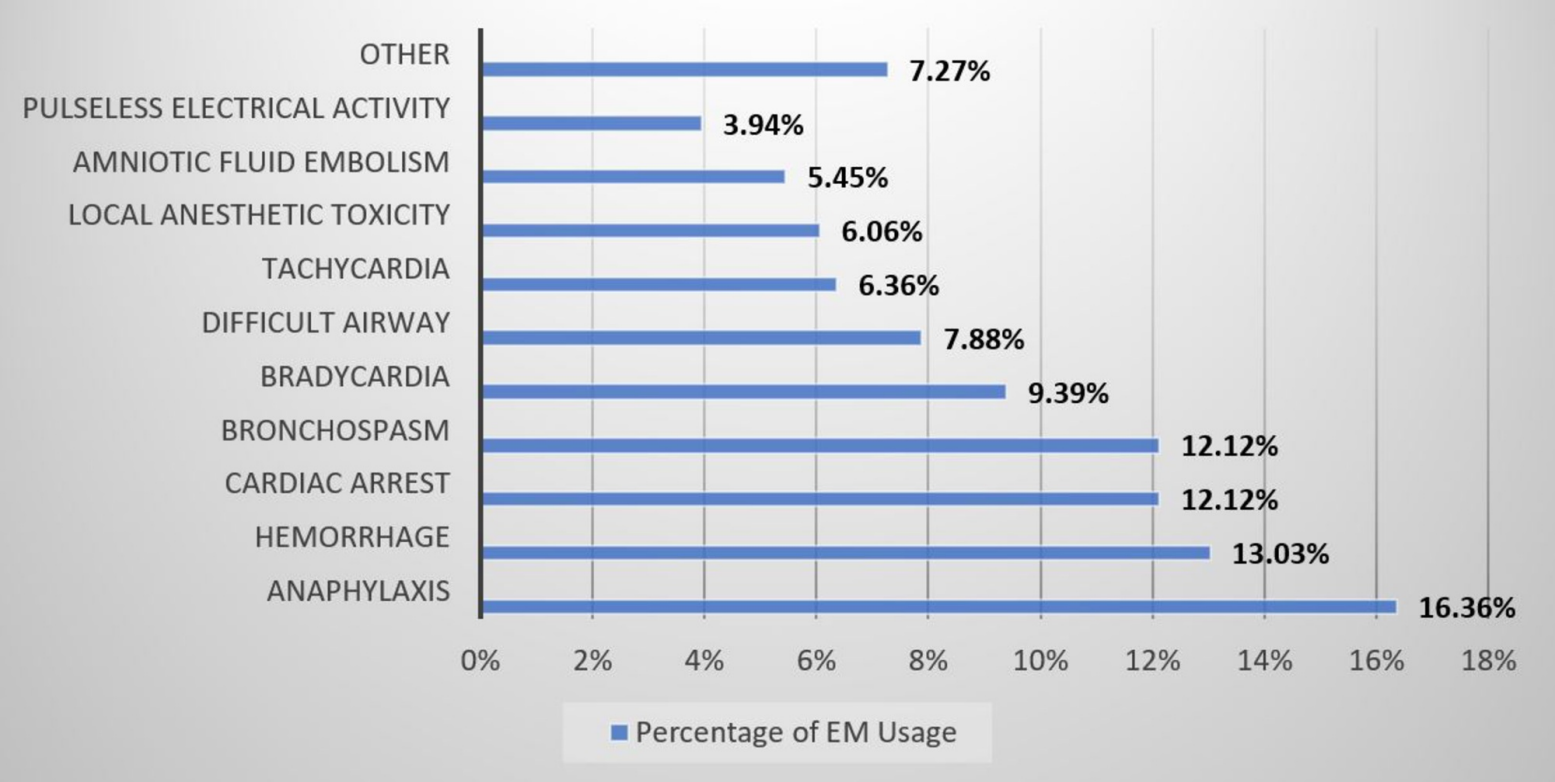
Emergency manual use reports for clinical critical events by event type

The type of critical events for which EM was most commonly used is shown in Figure 1. Overall, respondents have used the EM most often for anaphylaxis (16.36%), followed by hemorrhage (13.03%), cardiac arrest (12.12%), and bronchospasm (12.12%).

Others included the following: oxygen failure, pneumothorax, power failure, transfusion reaction, venous air embolism, hypotension, hypoxemia, delayed emergence, fire, malignant hyperthermia, and total spinal anesthesia.

The experienced EM users’ opinion about the best locations of EM used during critical events is shown in Table 2. Above 92% of respondents stated they used the EM in the anesthesia station of the operating room during critical events. 

**Table 2:** Location of EMs used during critical events EM, emergency manual

Location of emergency manuals	Percentage of participants
EM in the anesthesia station of operating room	92.11%
EM in the code cart	7.02%
EM in the anesthesia office	0.88%

The preferred reader when EM was used during critical events is shown in Table 3. Near fifty eight percentage of respondents believed that the senior physician should read EM during critical events, while only 7.02% thought the operating room nurse should read the EM during critical events. 

**Table 3:** Preferred reader of emergency manuals during critical events

Reader of emergency manuals during critical events	Percentage of participants
Senior physician	57.89%
Junior physician	23.68%
Anesthesia nurse	11.40%
Operating room nurse	7.02%

The opinion of respondents on how EMs benefit crisis management is shown in Table 4. Most respondents felt that EMs improved their confidence during crisis management, enhanced team cooperation during crisis management, and made crisis management more organized. 

**Table 4:** Benefits of emergency manual usage during critical events

	EMs improved confidence during crisis management	EMs made crisis management more organized	EMs enhanced team cooperation during crisis management
Yes	97.37%	100.00%	99.12%
No	2.63%	0.00%	0.88%

## Discussion

Despite the widespread usage of EMs and well-documented benefit of their use during critical events, there is currently no standardized protocol for EM usage. There is also a lack of research focused on maximizing its efficiency. To our knowledge, no study has been performed to assess the best location for EMs and the preferred reader for EMs during critical events. 

Our study showed that the best location of EMs used during critical events was in the anesthesia station of the operating room. The result supported Stanford’s recommendation about the location [2]. Easy access to EMs by the providers is essential, especially during a critical event when every second count. The location of the EM should maximize efficiency by minimizing the time spent retrieving it, and perhaps the greatest way to minimize the retrieval time is to have the EMs located in the operating room itself.

Additionally, our study showed that the preferred reader of EMs during critical events is the senior physician, which suggests that the reader should be an experienced team member with a high degree of leadership over the clinical team. 

It is generally believed that the senior physician has ultimate responsibility for the clinical outcomes in China. Therefore, they believe the senior physician should be both the reader and the leader. The distinction between the reader and the leader is important. The reader may temporarily assume a leader-like role, as the other team members directly follow the instructions being read, but the reader holds no direct authority in making the final clinical decision. The leader, in contrast, oversees the final decision-making and holds direct clinical responsibility for the clinical outcome. By assigning the reader role to the leader, any adverse outcome caused by incorrectly reading the EMs is ascribed to the same individual responsible for taking ownership of that adverse outcome. Careful assignment of the reader role to a team member who is also a leader, thus, is crucial because the reader’s actions have direct implications on the process of EM usage and the consequences--whether positive or negative that follow the critical event.

While our primary research goal was to determine the best location and reader of EMs, our results also provided evidence that most anesthesiologists realize the benefits of using EMs, which include enhancing provider confidence when responding to critical events, increasing the organization of crisis management, and enhancing team cooperation (Table 4). These results further reiterate the importance of EMs in crisis management and the potential benefits of standardizing their use. EMs use reports for the critical events by event type in this study were similar to the results from the previous study [8]. Some participants in these studies were from the same hospitals.

Our results may be applicable to the development of a standardized protocol for EMs usage which, as suggested by our survey, maximizes efficiency by having the EMs located in the operating room and assigning the reader role to the senior physicians if available. 

Our study is based on the opinion of anesthesiologists with several years of clinical experience in responding to emergency care situations; more than 42% of respondents have been practicing medicine for over 10 years, and more than 70% have greater than five years of experience. As such, their opinion on utilizing EMs may be regarded as an expert, since it is likely grounded on substantial first-hand experience navigating critical events with and without the use of EMs. 

Limitations

It is difficult to conduct a control study to answer these questions, given the spontaneous nature of such critical events that require the use of EMs. Our results are also limited by the sample size, which should be substantially larger to accurately represent the average opinion of anesthesiologists in the U.S. Additionally, there is always the possibility of bias in the participants which may be influenced by many factors including age, ethnicity, and geographical location. We acknowledge that more research must be done to determine whether this location and reader role definitively maximizes efficiency more than alternative options; perhaps even those which have not been traditionally considered. 

Our survey asked participants to recall critical events in the past year. Due to the nature of this study, accuracy and honesty cannot be verified, and some respondents might have forgotten to recall certain events. Given that providers who use cognitive aids may be more likely to report events, there is a potential for selection bias as well. 

While this study demonstrates an increased utilization of EM in the OR, there is no evidence of improvements in clinical outcomes. The real value of EMs depends on whether it results in improved patient outcomes (such as, by reducing morbidity and mortality), which is a topic that should be explored in future research.

## Conclusions

Our study indicates that anesthesiologists prefer the EMs to be placed in the operating room and that the senior physician should be the reader of EMs. These results could be used as evidence to develop a national standardized protocol for the implementation and usage of EMs. Further research is required to evaluate whether EMs improve patient outcomes.
